# The Effect of Carbon Monoxide Co-Adsorption on Ni-Catalysed Water Dissociation

**DOI:** 10.3390/ijms141223301

**Published:** 2013-11-26

**Authors:** Abas Mohsenzadeh, Anders Borjesson, Jeng-Han Wang, Tobias Richards, Kim Bolton

**Affiliations:** 1 School of Engineering, University of Borås, Borås SE 501-90, Sweden; E-Mails: anders.borjesson@combine.se (A.B.); tobias.richards@hb.se (T.R.); 2 Department of Chemistry, National Taiwan University, No. 88, Sec. 4, Ting-Chow Rd, Taipei 11677, Taiwan; E-mail: jenghan@ntnu.edu.tw

**Keywords:** water adsorption, water dissociation, nickel, water gas shift reaction, CO, H_2_O, DFT

## Abstract

The effect of carbon monoxide (CO) co-adsorption on the dissociation of water on the Ni(111) surface has been studied using density functional theory. The structures of the adsorbed water molecule and of the transition state are changed by the presence of the CO molecule. The water O–H bond that is closest to the CO is lengthened compared to the structure in the absence of the CO, and the breaking O–H bond in the transition state structure has a larger imaginary frequency in the presence of CO. In addition, the distances between the Ni surface and H_2_O reactant and OH and H products decrease in the presence of the CO. The changes in structures and vibrational frequencies lead to a reaction energy that is 0.17 eV less exothermic in the presence of the CO, and an activation barrier that is 0.12 eV larger in the presence of the CO. At 463 K the water dissociation rate constant is an order of magnitude smaller in the presence of the CO. This reveals that far fewer water molecules will dissociate in the presence of CO under reaction conditions that are typical for the water-gas-shift reaction.

## 1. Introduction

The water gas shift (WGS) reaction, CO + H_2_O → CO_2_ + H_2_, is important in many industrial processes, including methanol synthesis and production of hydrogen for use in, e.g., fuel cells. It is also one of the most important reactions in gasification, where carbonaceous materials are converted to a gaseous product that can be used to produce energy or other desirable chemicals [1–5]. The efficiency of the WGS reaction is enhanced in the presence of transition metal catalysts such as nickel, which is widely used due to its high heat conductivity, high catalytic conversion and its capability to be manufactured in different shapes [6–9].

Due to its industrial significance, several experimental and computational investigations have focused on the WGS reaction. Bond *et al.* proposed a modified route for the gold-catalyzed WGS reaction mechanism by thermal decomposition of a carboxyl species [10]. Steady-state WGS kinetics were determined on ceria-supported Pd, Pt and Rh catalysts by Gorte *et al.*; they found that the ceria structure significantly affects the results [11]. Shekhar *et al.* have investigated the promotional effect of alkali additives (Na, Li and K) on the WGS reaction for Pt/Al_2_O_3_ and Pt/TiO2 catalysts. They showed that the active platinum remains in the metallic state and that the promotion by alkali is due the modification of the support properties [12]. A density functional theory (DFT) study together with experimental data for the WGS reaction catalyzed by Pt were provided by Gokhale *et al.* They predicted that that the most significant reaction channel proceeds via a carboxyl intermediate while formate acts only as a spectator species [13]. Furthermore, Cordeiro *et al.* studied the role of the step sites in the WGS reaction catalyzed by Cu and found that the associative route through the carboxyl intermediate assisted by co-adsorbed OH is favored in the presence of steps [14].

The four mechanisms that have been suggested for the WGS reaction are the redox, formate, associative and carbonate mechanisms [15–27]. Previous first principles calculations showed that the most probable reaction mechanisms are the carboxyl and redox mechanisms, and that the rate-limiting step is water dissociation [14,15,28–33].

The catalytic dissociation of water is also important in many other industrial processes, such as steam methane reforming (SMR; CH_4_ + H_2_O → CO + 3H_2_) where nickel is frequently used as catalyst. The SMR reaction involves the conversion of a hydrocarbon fuel (or an alcohol) into another fuel containing higher heating value. The SMR reaction is widely implemented for production of hydrogen or other useful products [34–36]. The water dissociation is believed to be one of the rate controlling elementary reaction steps in the SMR reaction [37].

The catalytic dissociation of water has been widely studied using both experimental and computational techniques. For example, hydroxyl radical production following Ni-catalyzed water dissociation has been investigated experimentally by Keiser *et al.* [38]. The hydroxyl radicals were monitored using laser-induced fluorescence and the barrier for their desorption was estimated at different temperatures and pressures. Fajín *et al.* studied water dissociation on metal surfaces using DFT. They predicted that the nickel surface could be effective for catalyzing water dissociation, that the activation barrier is 0.71 eV and the reaction energy is −0.37 eV [33]. The binding energies, preferred adsorption sites and configurations for water and its dissociation products (OH and H) were determined over a number of surfaces, including Ni(111), by Phatak *et al.* They found that dissociation of H_2_O to OH and H is exothermic on Ni(111) and the activation and reaction energies are 0.96 eV and −0.2 eV, respectively [39]. Several studies have also focused on fundamental aspects of water dissociation on metal surfaces, such as the vibrational modes of the molecular and dissociated water [40–44].

In addition to the WGS reaction discussed above, interaction of CO with transition metal surfaces is of importance in many catalytic reactions, such as the oxidation of carbon monoxide, CO methanation and Fischer-Tropsch synthesis [45]. These interactions, including the interactions between adsorbed CO and H_2_O, have been investigated over Ni(111) [46–48]. For example, high resolution electron energy loss spectroscopy (HREELS) measurements by Ellis *et al.* revealed strong interaction between H_2_O and CO adsorbed on Ni(100), where the co-adsorbed CO changes the water OH stretching properties [49]. DFT calculations by Lin *et al.* yielded a reaction energy of −0.11 eV and an activation barrier of 0.79 eV for water dissociation in the presence of CO [50]. This reaction energy is less exothermic than those obtained in the absence of CO (−0.37 and −0.2 eV, see above) and the activation barrier lies between the values obtained in the absence of CO (0.71 and 0.96 eV). These, and other co-adsorption studies of reactive chemical species on transition-metal catalysts, [51–54] are essential for a more complete understanding of heterogeneous catalysis.

The present contribution extends these previous investigations by using DFT to perform a comparative study of the dissociation of water on a Ni(111) surface in the absence and presence of co-adsorbed CO. This is the first time that these systems have been studied using the same models and computational methods, and is important since, if the co-adsorbed CO affects the reactant, transition state or product relative energies or vibrational frequencies, then the water dissociation rate will depend on the presence of CO. For example, the rates may be different for the reaction in a CO-rich WGS and when water dissociates in other processes when CO is not present. In addition, molecular-level understanding of the water dissociation mechanism which, as discussed above, is a key elementary step in a number of important reactions such as WGS and SMR, will assist in designing more effective catalysts.

## 2. Results and Discussion

### 2.1. Adsorption Sites and Energies

**Figure 1.**
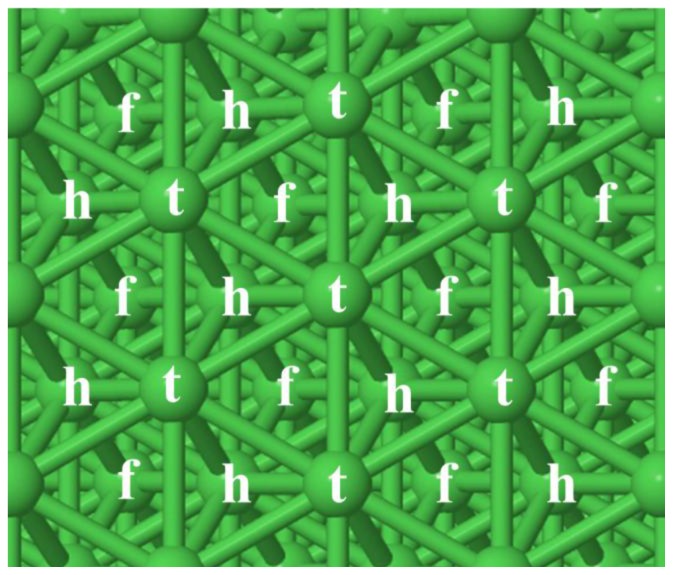
Top (t), hollow hcp (h) and hollow fcc (f) sites on the Ni(111).

**Figure 2.**
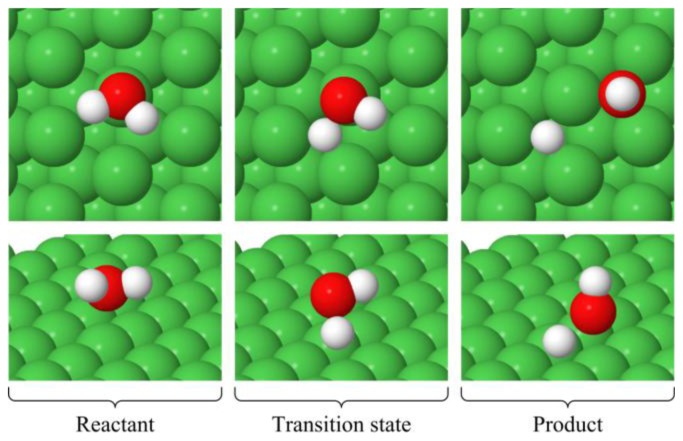
Minimum energy structures of the reactant, transition state and product for the H_2_O^*^ → OH^*^ + H^*^ reaction.

**Figure 3.**
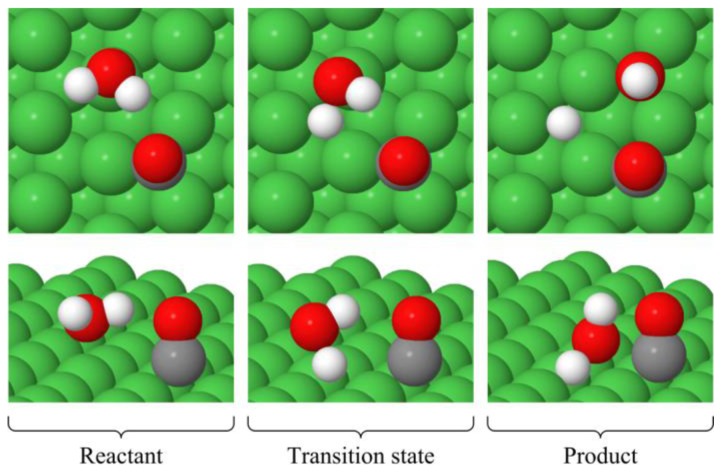
Minimum energy structures of the reactant, transition state and product for the H_2_O^*^ + CO^*^ → OH^*^ + H^*^ + CO^*^ reaction.

The top (t), hollow hcp (h) and hollow fcc (f) sites on the Ni(111) surface are shown in Figure 1. Geometry optimization of the reactants and products was performed on the sites that have previously been shown [50] to yield the lowest energy structures (*i.e*., the most favorable sites). The lowest energy structures in the absence and presence of CO are shown in Figures 2 and 3, respectively, and details of these structures are shown in Table 1. The * in these figures and table indicates that the specie is adsorbed on the surface.

In the absence of CO, the preferred adsorption site for water is the top site (t) via the O atom. The adsorption energy is −0.27 eV (−0.36 eV without zero point vibrational energy [ZPVE] correction). Both of the OH and H products favor the hollow fcc site (f) and, similarly to the water molecule, the OH is adsorbed via the O atom. The adsorption energy of these products is −5.83 eV (−6.23 eV without ZPVE correction).

The presence of the CO does not affect the preferred adsorption sites. The favored adsorption site for water is the top site (t) via the O atom and for carbon monoxide it is the hollow fcc site (f) via the C atom. The adsorption energy is −2.32eV (−2.38 eV without ZPVE correction). All of the products prefer the hollow fcc site (f), where the OH is adsorbed via the O atom and CO via the C atom. The adsorption energy is −7.63 eV (−7.99 eV without ZPVE correction).

The calculations performed for the higher surface coverages yielded similar results to those presented above. For example, for 1/4 monolayer (only water) and 1/2 monolayer (co-adsorbed water and CO) coverages, the reactant adsorption energies are −0.20 eV (−0.26 eV without ZPVE correction) and −2.43 eV (−2.35 eV without ZPVE correction), respectively.

Although the presence of the CO does not affect the preferred adsorption site, it does affect the reactant geometry. In the absence of the CO the lengths of the O–H bonds in the water molecule are almost the same (0.98 Å) while, in the presence of CO, the O–H bond closest to the CO molecule is 0.015 Å longer than the other bond. This larger bond length is probably due to interactions between this H atom and the CO molecule (the distance between this H and the O on the CO is just 1.88 Å), although the charge density on this H atom is the same as the charge density on the other H atom. This is also reflected in the vibrational frequencies where the asymmetric stretching mode is lowered by 200 cm^−1^ (from 3612 to 3412 cm^−1^ in the absence and presence of CO, respectively). The presence of the CO does not have a significant effect on the OH product geometry or vibrational frequency.

The co-adsorbed CO also affects the distance between the reactant and products with the Ni surface (defined as the shortest distance between any atom of the adsorbate and any metal atom on the surface). The distance between the H_2_O and the surface decreases from 2.157 Å to 2.114 Å when the CO is present, for OH the decrease is from 1.950 Å to 1.923 Å, and for H the decrease is from 1.655 Å to 1.646 Å. The reason that the presence of the CO molecule decreases the height of the reactant and products above the surface is probably due to electron transfer from the CO to the Ni surface. In the presence of the CO the total charge density on the uppermost Ni atoms is 0.63 e more than in the absence of the CO. This increases the interaction strengths (and hence decreases the bond lengths) between the surface and the adsorbants, which is also seen by an increase in the charge density on the H_2_O, OH and H adsorbants by 0.01, 0.04 and 0.03 e, respectively.

### 2.2. Transition States and Reaction Energies

Table 2 shows the data of the transition states together with reaction rate constants at 463 K, which is typical for low-temperature processes that include the WGS reaction [55]. The activation energy (E_a_) without co-adsorbed CO is 0.75 eV (0.96 eV without ZPVE correction) and in the presence of CO it increases to 0.87 eV (1.09 eV without ZPVE correction), respectively. The activation energy in the absence of CO is in agreement with that obtained by Fajín *et al.* (0.71 eV). Similarly, the value determined in the presence of CO is similar to the value of 0.79 eV reported by Lin *et al.* The same trend is found for higher coverages. For the 1/4 and 1/2 monolayer surfaces the activation barrier in the absence of CO is 0.69 eV (0.87 eV without ZPVE correction) and in the presence of CO it is 1.18 eV (1.39 eV without ZPVE correction). Hence, the presence of CO increases the activation energy at both surface coverages.

The length of the breaking O–H bond is not significantly influenced by CO co-adsorption, and its (imaginary) vibrational frequency is increased by only 20 cm^−1^. This means that the reaction barrier is slightly narrower in the presence of the CO. However, this effect is not as significant as it is for the water reactant, where the presence of the CO decreased the asymmetric vibrational mode frequency by 200 cm^−1^.

Unlike the length and frequency of the breaking bond, the reaction rate constant changes considerably when CO is present, decreasing from 2.03 × 10^4^ s^−1^ to 1.76 × 10^3^ s^−1^. The rate constant in the absence of CO is smaller than the value of 7.1 × 10^4^ s^−1^ obtained by Fajín *et al.* [33]. This may be due to the convergence criteria, since repeating the above calculation, but with coarser convergence criteria of 10^−5^ eV for the total energy and 10^−2^ eV/Å for the forces acting on ions, yields the same result as that reported by Fajín *et al.*

The reaction energy (E_react_), which is the difference between the product and reactant energies, is significantly affected by the presence of co-adsorbed CO. In the absence of CO E_react_ = −0.41 eV (−0.30 eV without ZPVE correction) which is 0.24 eV more exothermic than the reaction energy in the presence of CO, which is −0.17 eV (−0.05 eV without ZPVE correction). The reaction energy calculated in the absence of CO is similar to that obtained by Fajín *et al.* (−0.31 eV). Similarly, the calculated reaction energy in the presence of co-adsorbed CO is similar to the value of −0.11 eV obtained by Line *et al.* [33,50].

**Figure 4.**
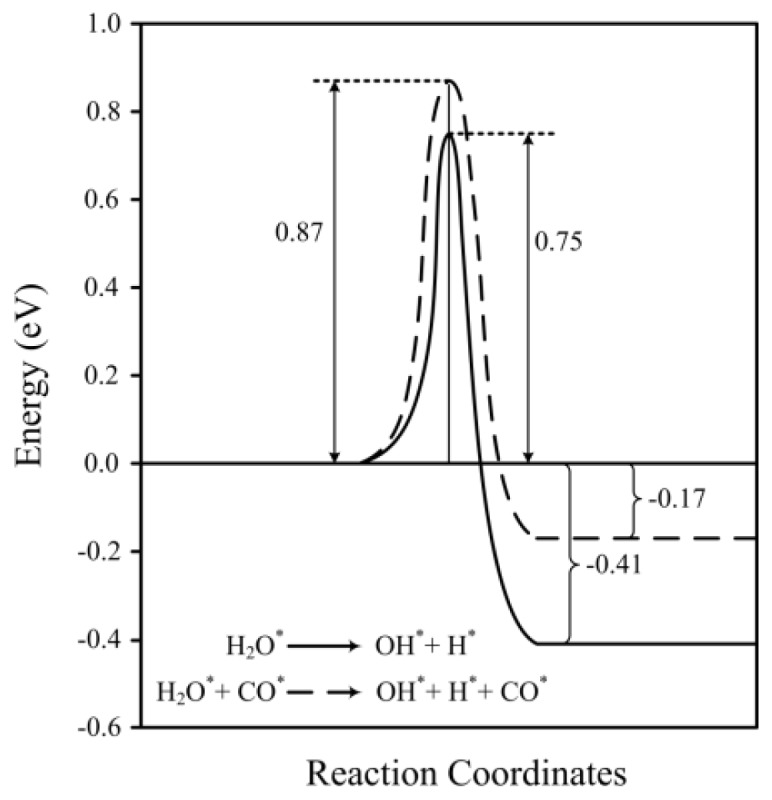
Reaction profiles for the water dissociation with (**dashed line**) and without (**solid line**) co-adsorbed CO.

A comparison of the reaction profiles with and without adsorbed carbon monoxide is shown in Figure 4. As discussed above, the co-adsorbed CO increases the activation barrier by 0.12 eV and decreases the exothermicity by 0.24 eV. A possible reason for the larger activation energy is that the CO (and Ni surface) induces a larger change in the structure of the water molecule when going from reactant to transition state. To investigate these we performed single point energy calculations on the water molecule (in vacuum) in its reactant and transition state structures. It was seen that this does not explain the trends seen in Figure 4, since the energy of the water molecule in the transition state structure is lower than the energy in the reactant structure, and hence it is changes in the CO, Ni surface or interactions between the water-CO-Ni that lead to the increase in energy at the transition state. Similarly, calculations comparing the energies of the reactant water and product H and OH structures showed that this cannot explain the decrease in exothermicity in the presence of the CO. In fact, when comparing the H_2_O and (H + OH) energies the reaction is endothermic when using the structures from the both systems (in the absence and presence of the CO).

These calculations were repeated but where the CO molecule was included (together with the water molecule). This was done to ascertain whether it is changes in the CO molecule and/or interactions between the H_2_O and CO that leads to an increase in the transition state energy compared to the energy of the reactant. Once again, the H_2_O-CO energy (in the vacuum) of the transition state structure was lower than the energy of the reactant structure, and the reaction is more exothermic than that when using the structures from the system that does not contain CO (thus opposite to what is observed in Figure 4).

Hence, it is the interplay between all three components—the H_2_O, CO and Ni surface—that leads to the reaction profiles seen in Figure 4. As discussed above, the presence of the CO on the surface leads to stronger interactions between the Ni surface and the H_2_O reactant and H and OH products. The larger activation energy and decreased exothermicity indicates that the stability induced by the surface in the presence of CO is larger for the reactant than for the transition state and product structures.

**Figure 5.**
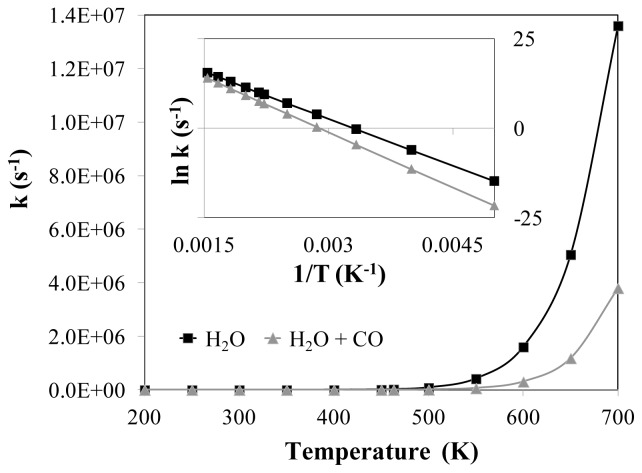
Temperature dependence of the reaction rate constant for the water dissociation reaction with and without co-adsorbed carbon monoxide. The Arrhenius format of the data is shown in the inset.

Figure 5 shows the effect of temperature on the reaction rate constant. The data is also shown in its Arrhenius form in the inset to the figure. The difference in reaction rate constants in the absence and presence of CO increases with increasing temperature, showing that it becomes even more important to consider the effect of CO co-adsorption at higher temperatures.

## 3. Methods and Models

The calculations were performed with the Vienna *ab initio* simulation package (VASP) [56–59] using spin polarized DFT. The Perdew-Burke-Ernzerhof generalized gradient approach (GGA-PBE) [60] to the exchange-correlation potential was implemented and the projector-augmented wave method (PAW) [61,62] was applied to the basis set to account for the effect of the core electrons in the valence electron density. A 600-eV cutoff for the plane waves expansion was applied and a 4 × 4 × 1 Monkhorst-Pack grid of *k*-points [63] was used for the numerical integration in reciprocal space. As shown previously [50] smaller Brillouin zone (BZ) sampling intervals (5 × 5 × 1 and 6 × 6 × 1) and higher cutoff energies (700 and 800 eV) show insignificant differences in the energies of the optimized structures (less than 0.01 eV). Hence, the cutoff and Monkhorst-Pack grid used here yield converged results.

The surface orientation, as well as steps and defects on the surface, could affect its catalytic properties and reactions energies [14,64]. The face-centered cubic (fcc) nickel, Ni(111), is the most stable Ni surface and is therefore commonly used in computational studies of heterogeneous catalytic reactions [65–69]. This surface was also used in the present work, where periodic boundary conditions were imposed in two directions to model a semi-infinite crystal surface. Tests showed that a Ni(111) surface containing 4 × 4 unit cells in each layer, and with five layers that are separated by an equivalent volume of vacuum in the surface perpendicular direction, yield converged results in a computationally tractable time. The two bottom layers of the slab were fixed to maintain the bulk crystal structure, and the three upper layers were free to relax. This periodic box size, which yields a 1/16 monolayer coverage for the water in the absence of CO and a 1/8 monolayer for the co-adsorbed CO and water, prevents interactions between the surface atoms and adsorbates with their periodic images. Higher surface coverages, including 1/4 and 1/2 monolayer, were also investigated to elucidate if the trends reported here are sensitive to the surface coverage.

The conjugate-gradient (CG) method was used to obtain the geometry optimized structures of the adsorbates on the surface. The convergence criteria were 10^−6^ eV for the total energy and 10^−3^ eV/Å for the forces acting on the ions.

Transition states were identified using an improved version of the nudged elastic band (NEB) method, called climbing-image NEB (CI-NEB) [70,71]. In this method, the lowest energy reactant and product configurations are selected as the initial and final states, and 6 images were placed along the minimum energy path (MEP). A −0.5 eVÅ^−2^ spring force constant between images was used. Due to computational constraints, a smaller set of *k*-points (2 × 2 × 1) and a lower energy cutoff (400 eV) was used to relax all the images until the maximum force acting on an atom was less than 0.01 eV. Single point energy calculations at the transition state using 4 × 4 × 1 *k*-mesh and 600 eV cut off showed that the activation energy differs from that obtained with the less accurate settings by at most 0.02 eV.

Vibrational frequencies were calculated at all stationary points to ensure that they were minimum energy (zero imaginary frequencies) or transition states (one imaginary frequency) geometries, as well as to determine the zero point vibrational energies (ZPVEs) and partition functions. The frequencies were determined by diagonalizing a finite difference construction of the Hessian matrix using displacements of 0.01 Å (only the adsorbates were allowed to move).

The adsorption energies (E_ads_) of the reactants and products were calculated from Equation (1).

(1) $${\text{E}}_{\text{ads}}={\text{E}}_{\text{surf}+\text{adsorbate}}-{\text{E}}_{\text{surf}}-{\text{E}}_{\text{adsorbate}}$$

where E_surf_ is the total energy of the Ni(111) surface, E_adsorbate_ is the total energy of the isolated, geometry optimized adsorbate(s) in the gas phase and E_surf + adsorbate_ is the total energy of the surface-adsorbate(s) system. Results of E_ads_ where ZPVE corrections are excluded ( ${\text{E}}_{\text{ads}}^{\text{e}}$) and included ( ${\text{E}}_{\text{ads}}^{\text{o}}$) are given below for the sake of completeness and to show the importance of this correction.

The water dissociation rate constant (*k*) was estimated using transition state theory [72], *i.e.*, Equation (2).

(2) $$k_{\text{TST}}=\left(\frac{k_{B}T}{h}\right)\;\left(\frac{q^{\#}}{q}\right)\;{\text{e}}^{\frac{-{\text{E}}_{\text{a}}}{k_{B}\text{T}}}$$

where *k_B_* is Boltzmann’s constant, *T* is the absolute temperature, *h* is Planck’s constant and E_a_ is the activation energy from the ZPVE corrected energies. *q* and *q*^#^ are the partition functions for the reactant and the transition state respectively. Similarly to previous studies [14,33,73], the partition functions have been calculated assuming harmonic vibrations. Although this approximation may well affect the quantitative results presented here, it is not expected to affect the trends.

The effect of the CO on the reactant, transition state and product geometries was analyzed using the atomic charge densities. These calculations were performed using 2 × 2 unit cells in each layer, since these cells yield the same geometric and energetic trends as the larger unit cells. The charge density on each ion in the relaxed structure is calculated by integrating the valence charge density within the Wigner-Seitz spheres around each atom. This radius is selected such that the total volume over all atoms is approximately 100% and that the ratios of the atomic radii is equal to that of the ionic radii [74]. The present calculations use a 8 × 8 × 1 *k*-point meshes and Wigner-Seitz radii equal to 1.4 Å for Ni, 1.29 Å for carbon, 1.11 Å for oxygen and 0.7 Å for hydrogen. Altering these radii by up to 10% does not change the trends reported here.

## 4. Conclusions

The effect of CO co-adsorption on water dissociation over the Ni(111) nickel surface has been studied using DFT calculations. The results show that the co-adsorption of CO alters the geometry of the adsorbed reactant water molecule. The O–H bond that is closest to the CO is lengthened and weakened. In addition, the distance between the reactants and products with the surface decreases in the presence of the co-adsorbed CO. These changes result in a dissociation energy that is 0.24 eV less exothermic in the presence of the CO.

The results also show that the activation energy for water dissociation increases by 0.12 eV in the presence of the co-adsorbed CO. In addition, the breaking O–H bond at the transition state has a slightly larger imaginary vibrational frequency in the presence of the co-adsorbed CO. These changes (including changes in the reactant geometries and vibrational frequencies) lead to a considerable decrease in the rate constant when CO is present. At typical low-temperature process conditions of 463 K, the rate constant in the presence of CO is approximately twelve times smaller than in the absence of CO, and this difference increases with increasing temperature. Hence, it is important to account for co-adsorbed CO when Ni-catalyzed water dissociation occurs in a CO-rich environment.

## Figures and Tables

**Table 1.** Adsorption energies (eV), vibrational frequencies (cm^−1^) and structural parameters (Å) of reactants and products with and without co-adsorbed carbon monoxide. a

Species	Adsorption site	${\text{E}}_{\text{ads}}^{\text{e}}$	${\text{E}}_{\text{ads}}^{\text{o}}$	Vibrational frequencies	d_surf-mol_^b^	Bond length c
H_2_O^*^	t	−0.36	−0.27	3723, 3612, 1558, 489, 427, 227, 172, 122, 84	H_2_O: 2.157	O–H_a_: 0.979; O–H_b_: 0.978
OH^*^+H^*^	OH: f; H: f	−6.23	−5.83	3712, 1220, 952, 776, 546, 502, 387, 290, 248	OH: 1.950; H: 1.655	O–H_a_: 0.973
H_2_O^*^+CO^*^	H_2_O: t; CO: f	−2.38	−2.32	3721, 3412, 1662, 1566, 737, 516, 379, 349, 322, 306, 226, 190, 149, 128, 76	H_2_O: 2.114; CO: 1.903	O–H_a_: 0.990; O–H_b_: 0.975; C–O: 1.213
OH^*^ + H^*^ + CO^*^	OH: f; H: f; CO: f	−7.99	−7.63	3704, 1761, 1253, 972, 740, 567, 520, 415, 395, 322, 299, 277, 246, 166, 122	OH: 1.923; H: 1.646; CO: 1.872	O–H_a_: 0.974; C–O: 1.195

a For the adsorption energies (E_ads_); “^e^” and “°” denote the uncorrected and ZPVE-corrected values, respectively;

b Shortest distance between any atom of the adsorbate(s) and any metal atom on the surface;

c Letter “a” shows the O–H bond nearest the CO and “b” the other O–H bond.

**Table 2.** Activation energies (eV), vibrational frequencies (cm^−1^), reaction rate constant at 463 K (s^−1^), reaction energy (eV), length of the breaking OH bond at the transition state and its imaginary frequency (cm^−1^), with and without co-adsorbed carbon monoxide. a

Species	${\text{E}}_{\text{a}}^{\text{e}}$	${\text{E}}_{\text{a}}^{\text{o}}$	Vibrational modes	*k*	${\text{E}}_{\text{react}}^{\text{e}}$	${\text{E}}_{\text{react}}^{\text{o}}$	d_O–H_	Imaginary frequency
H_2_O^*^ → OH^*^ + H^*^	0.96	0.75	3653, 837, 748, 677, 432, 393, 167, 71	2.03×10^4^	−0.30	−0.41	1.559	797
H_2_O^*^ + CO^*^ → OH^*^ + H^*^ + CO^*^	1.09	0.87	3623, 1726, 921, 765, 690, 465, 400, 377, 324, 282, 162, 147, 136, 105	1.76×10^3^	−0.05	−0.17	1.560	817

a For the activation energies (E_a_), “^e^” and “°” denote the uncorrected and ZPVE-corrected values, respectively.
